# Re-development of mental health first aid guidelines for non-suicidal self-injury: a Delphi study

**DOI:** 10.1186/s12888-014-0236-5

**Published:** 2014-08-19

**Authors:** Anna M Ross, Claire M Kelly, Anthony F Jorm

**Affiliations:** Melbourne School of Population and Global Health, The University of Melbourne, Level 4, 207 Bouverie St, 3010 Parkville, Victoria Australia; Mental Health First Aid Australia, Level 6, 369 Royal Parade, 3052 Parkville, Victoria Australia; School of Psychology, Deakin University, Burwood, Victoria 3125 Australia

**Keywords:** Non-suicidal self-injury, Self-harm, Cutting, Mental health first aid, Helping behaviour, Mental health assistance

## Abstract

**Background:**

Up to 12% of Australian adults and almost one in five adolescents are estimated to have engaged in non-suicidal self-injury (NSSI) at some time in their life. Friends and family are most likely to notice signs of NSSI, but may be unsure how to intervene. Mental health first aid guidelines were developed in 2008 on how to do this through providing initial support and encouraging appropriate professional help-seeking. This study aims to re-develop the 2008 NSSI first aid guidelines to ensure they contain current recommended helping actions and remain consistent with the NSSI intervention literature.

**Methods:**

The Delphi consensus method was used to determine the importance of the inclusion of helping statements in the guidelines. These statements describe helping actions a member of the public can take, and information they should have, to help someone who is engaging in NSSI. Systematic searches of the available NSSI intervention literature were conducted to find helping statements. Two expert panels, comprising 28 NSSI professionals and 33 consumer advocates, rated the importance of each statement.

**Results:**

98 out of 220 statements were endorsed as appropriate helping actions in providing assistance to someone engaging in NSSI. These statements were used to form the updated mental health first aid guidelines for NSSI.

**Conclusion:**

The re-development of the guidelines has resulted in more comprehensive guidance than the original version (98 versus 30 statements containing helping actions). This substantial increase in endorsed statements adds detail and depth to the guidelines, as well as covers additional ways of providing guidance and support.

**Electronic supplementary material:**

The online version of this article (doi:10.1186/s12888-014-0236-5) contains supplementary material, which is available to authorized users.

## Background

Non-suicidal self-injury (NSSI) refers to injuries that are deliberately inflicted on oneself that are not intended to result in death [1,2]. The most common methods of self-injury are cutting, scratching, deliberately hitting body parts on a hard surface, punching, hitting or slapping oneself, biting and burning [3]. The Australian National Epidemiological Study of Self-Injury (ANESSI) found that 19.3% of Austalian adolescents and 11.9% of Australian adults have engaged in self-harming behaviours at some time in their life [3]. This prevalence has also been found to be higher in clinical populations, with research findings revealing that those experiencing anxiety, mood disorders and other mental health problems were 5.5 to 7.7 times more likely to self-injure over the previous 4 weeks, and that at least 21% of clinical psychiatric populations self-injure over a 6 month period [3–5].

People have reported engaging in NSSI for many reasons. The most common include the management of painful feelings, self punishment, and to communicate a message to others [3]. Contrary to popular belief, self-injury is rarely used as a means of seeking attention. The cuts and wounds inflicted through self-injury often cause people to feel intense shame and as a result are likely to be covered or hidden underneath clothing.

NSSI can be differentiated from suicidal behaviour through the intentions behind the injuring. The difference is considered to be based on the intent and the underlying emotions of the person engaging in these actions [6]. Those who are having suicidal thoughts and engaging in suicidal behaviour harm themselves with the intent of ending their life, whereas those who engage in NSSI do not intend to end their lives. In this article, suicidal behaviour has been separated from non-suicidal self-harm, as these behaviours require different first aid assistance, such as different intervention methods and different urgencies in obtaining professional help.

However, there may be an association between NSSI and suicide, with rates of suicide higher in those who self-injure [7–9]. Those engaging in self-injury have been found to be at higher risk for suicidal thoughts [3,10], with the risk of suicide increased by 30-100 times compared to the general population [7,11,12]. Furthermore, approximately half to two-thirds of persons who die by suicide have been found to have a history of NSSI [7,13,14]. Although NSSI is common and is associated with increased risk of suicide, most people who self-injure do not seek professional treatment, with less than 50% of persons reporting help seeking [3,15]. When help is sought, it is most likely to be from either family members or friends [10,16–20]. Family and friends are also most likely to notice possible signs of NSSI, such as fresh cuts or scars from cutting, but report having insufficient knowledge regarding what they can do to provide effective assistance for mental health problems [21].

Mental health first aid guidelines have been developed through a series of Delphi expert consensus studies to provide recommendations to members of the public on providing assistance to a person with a mental health problem, including depression, psychosis, substance use or eating disorders, or experiencing a mental health crisis, such as having suicidal ideation, experiencing a traumatic event or a panic attack, or engaging in non-suicidal self-injury [22–29]. These guidelines were used to inform the content of the 2^nd^ edition Mental Health First Aid (MHFA) course [2]. The programme teaches adult members of the public how to provide assistance to someone who has a mental health problem or is experiencing a mental health crisis, until appropriate professional assistance is received or the crisis resolves [30]. While intervening when someone has been engaging in NSSI is only briefly covered, this course has been found to be effective in providing the knowledge required to intervene and increasing helping behaviours [31].

The guidelines for assisting a person who engages in NSSI were developed in 2008. Because there are companion guidelines on first aid for a suicidal person, the term NSSI is used to make clear that these guidelines are for situations where there is no suicidal intent. Furthermore, this term is becoming dominant in the literature. As well as informing the content of MHFA training, these guidelines were made available online for the public to access. The guidelines were accessible through the National Health and Medical Research Council (NHMRC) Clinical Practice Guidelines Portal, and were also made available for free download from the MHFA website (https://mhfa.com.au/cms/guidelines). A study by Hart et al. [32] showed that users who download the guidelines do make use of them to assist in mental health first aid situations.

To ensure the guidelines reflect current evidence and best practice, re-development of these guidelines is required to update their content, to take into account the latest NSSI intervention research findings and recommendations from NSSI intervention experts. Re-development of the guidelines will also ensure that they meet the NHMRC Clinical Practice Guidelines Portal inclusion requirements, which require that guidelines be no more than five years old. The aim of this study was to use the Delphi methodology [33] to re-develop guidelines for members of the public providing first aid assistance to people who engage in deliberate non-suicidal self-injury. This method has been used to develop mental health first aid guidelines for a range of mental disorders, including the original version of the NSSI guidelines. This method was selected as it is considered a feasible and ethical approach to developing guidelines on a topic that is not amenable to evaluation in randomised controlled trials. The method allows the gathering of practice-based evidence from experts, so that their expertise can be conveyed to others. The method also allows expert consensus from panel members located in many countries to be obtained easily online.

## Method

The re-development of the guidelines was conducted in three stages: literature search, questionnaire development and Delphi consensus survey rounds.

### Literature search

A systematic literature search was conducted to find statements about how someone can help a person who is engaging in deliberate NSSI, including how to determine if someone is deliberately injuring themselves, how to offer short-term assistance to the person, and how to help them seek appropriate professional support. The literature searched included online materials, research publications, and self-help books.

Websites and online materials were searched using the Google search engines of English-speaking countries (Google.com, Google.com.au, Google.co.uk, Google.nz, Google.ca). The search terms 'self injury', 'self harm', ‘cutting’, as well as help (truncated to include terms such as ‘helping’ and ‘helped’) and ‘friend’ or ‘family’. The websites returned in the top 50 results from each search were reviewed. Overall, 146 unique websites were reviewed for potential first aid helping actions, with statements found on 57 of these sites.

The research literature was searched through PsycInfo and PubMed, with the terms ‘self harm’, ‘self injury’ or ‘NSSI’ searched for in the title and abstract, and ‘help’, ‘prevent’, ‘assist’, ‘support’ or ‘care’, as well as ‘friend’ or ‘family’ searched for throughout. Exclusion terms, comprising ‘cell suicide’, ‘assisted suicide’, and ‘suicide attack’ were also entered to improve relevance of results. Results were also limited to articles published after 2004, as the searches aimed to find new articles that not been included in the literature search in the earlier literature search for the original version of the guidelines. Search results returned 834 unique articles, with 22 considered relevant for review, and eligible statements found in 2 of these.

To locate relevant books, a search of Amazon.com was also conducted using the search terms ‘self harm’, ‘cutting’, ‘self- injury’, ‘help’ and ‘friend’ or ‘family’. Nine books were returned, with 5 of these considered relevant. These 5 books were purchased and read, with eligible statements found in all but one.

### Questionnaire development

Relevant helping statements that were found in the literature search, as well as the statements included in the previous Delphi questionnaires [25] formed the content of the first questionnaire. The statements included in the questionnaire were agreed upon by all three authors as being actionable by the first aider, as relevant to the role of a first aider, as well as being clear and non-ambiguous in its meaning. Examples of the types of statements included in the questionnaire include ‘The first aider should discuss their concerns with the person in a private place’ and ‘The first aider should let the person know the ways in which they are willing to help the person’. These statements were grouped into categories based on common thematic content. Statements were edited so that those with similar content were combined in order to reduce repetition throughout the questionnaire. Statements were also edited to improve clarity by systematic re-wording or elaboration through examples. This editing occurred in meetings of a working group, which were held to edit and develop a draft of the questionnaire, including its categories and structure of statements. The working group comprised the authors of this paper who are all researchers with previous experience in conducting research using the Delphi methodology and on MHFA training programmes.

The questionnaire was completed online through an online survey website, SurveyMonkey. Participants were given a two to three week time period to finish the questionnaire for each of the three rounds of the Delphi survey process. The questionnaires were able to be completed at times that were convenient to participants, and in multiple sessions if desired.

### Delphi consensus survey rounds

The consensus survey was conducted using the Delphi method [32]. The Delphi method involved identifying and recruiting panels of experts in the field of NSSI to rate the importance of helping statements. Statements that achieved substantial consensus regarding their importance for inclusion in the guidelines were considered as the recommended actions to take to help someone who is self-injuring.

Participants were recruited from developed English-speaking countries (Australia, Canada, Ireland, New Zealand United Kingdom, and the United States) to join one of two expert panels representing two areas of expertise: professionals or consumers. To be considered as having expertise in NSSI, panellists were required to have past personal experience in self-injuring, or professional experience working in the field of NSSI prevention and intervention (i.e. as a researcher, clinician, mental health nurse, social worker). Potential professional panellists were identified as experts through their involvement with NSSI research, prevention and intervention organisations, while potential consumer panellists were identified through their advocacy roles in NSSI prevention.

The profession panel was recruited through editorial boards of relevant academic journals and suicide prevention organisations. The heads of these boards and organisations were emailed an invitation to participate and a copy of the project’s plain language statement, asking that these be forwarded on to the relevant members. The academic journal editorial boards contacted included ‘Crisis’, ‘Suicide and Life-Threatening Behavior’ and the ‘Journal of Clinical Psychology’ special NSSI edition (November 2007, Volume 63, Issue 11). Professional panellists were also recruited through NSSI and suicide prevention organisations, such as the International Association of Suicide Prevention, Suicide Prevention Australia, the Australian Suicide Prevention Advisory Council, the American Foundation for Suicide Prevention, the American Association of Suicidology, the Canadian Association for Suicide Prevention, the Suicide Prevention Resource Center, the University of Oxford Centre for Suicide Research and Suicide Prevention Information New Zealand. Members of editorial boards and prevention organisations who were interested in participating were asked to give an expression of interest by contacting the authors and to provide an outline of their experience working with NSSI populations. Contact details for the authors were provided to potential participants in the Plain Language Statement sent to the editorial boards and prevention organisations. Interested persons who proved to have direct professional experience working in the field of NSSI prevention and intervention (i.e. as a researcher, clinician, mental health nurse, social worker) were added to the expert panel. Professionals were also asked to nominate any colleagues who they felt would also be appropriate panel members. The professional panel comprised 28 panellists, some of whom had multiple roles, including 7 professors and associate professors in psychiatry or psychology, 7 psychologists, 6 researchers, 6 social workers, 3 counsellors, 2 psychiatrists, 2 mental health service directors, and 3 who worked in other mental health support roles. This panel represented global professional opinions in NSSI prevention, demonstrated through their demographics presented in Table 1.

**Table 1:** **Participant characteristics (data collected in Round 1)**

	**n**	**Age range (years)**	**Median age (years)**	**% Female**	**# American**	**# Australian**	**# British**	**# Canadian**
Mental health professionals	28	28-69	40	75	13	10	3	2
Consumers	33	18-71	32	91	13	16	2	2

The consumer panel was recruited through depression and mental disorder advocacy organisations, including *beyondblue: the national depression initiative* (Australia), Depression and Bipolar Support Association (United States), National Alliance of Mental Illness (NAMI) (United States), Depression Alliance (United Kingdom), and Depression Support Network (New Zealand). Email invitations and plain language statements were emailed to the advocacy group coordinators for the information to be forwarded to the group members. Consumers who had written websites that offered support and information to other consumers, as well as promoted recovery from NSSI, were also identified as potential panellists and were invited to participate through email invitation. Consumers who were interested in participating were asked to give an expression of interest by contacting the authors and providing an outline of their first-hand experiences of NSSI. Contact details for the authors were provided to potential participants in the Plain Language Statement sent in the email invitations. Interested persons who claimed to have past personal experience in self-injuring and were comfortable reflecting on these experiences were added to the expert panel. Consumers were also asked to nominate anyone they knew who they felt would also be appropriate panel members. Thirty-three NSSI consumer advocates were recruited to this panel, with demographic characteristics also included in Table 1.

The outcome for each item was determined using the following criteria:

1. Statements that were rated as essential or important by 80% or more of the members in both panels were endorsed as helping actions to be included into the guidelines.
2. Statements were re-rated in a subsequent round of the questionnaire if:
  - Statements were rated as essential or important by 70-79.9% of the panel members
  - Statements were rated as essential or important by 80% of more of one panel, but less than 80% by the other panel
3. Statements that were rated as essential or important by less than 70% of both panel members were excluded.

In Round 1, panel members were also asked to provide feedback through a textbox at the end of each section of the questionnaire. This feedback textbox was intended for use by panellists to suggest helping actions that were not covered in the questionnaire, but generally panellists used the textboxes to provide rationales for their ratings. The comments made were reviewed by the working group. Suggestions that contained novel ideas were used to create new helping statements to be included in the subsequent Round 2 questionnaire. Also, statements that received feedback suggesting ambiguity in the interpretation of its meaning were re-phrased to make them clearer and included in Round 2. Statements from Round 1 that met the criteria to be re-rated were also included in the Round 2 questionnaire.

The third and final questionnaire was comprised of new statements that were developed from Round 1 feedback and presented for the first time in Round 2, but required re-rating in a further round. Items that still did not achieve consensus after being re-rated were rejected from inclusion in the guidelines.

Following each of the three rounds, each panellist was sent a report containing a summary of the results from the previous round. The report included a list of the statements that had been endorsed for inclusion in the guidelines, as well as a list of the statements that had been rejected from inclusion. The statements to be re-rated in the subsequent round were also included, with the report personalised to include the individual panellist’s rating for each statement, as well as a table summary of each panel’s ratings for the statement.

The statements that were endorsed across the three survey rounds were compiled. These statements were then used to form the guidelines, with working group meetings held to finalise structure and wording. The final draft copy of the guidelines was then disseminated to panellists for their final comment on the document.

## Results

Participation of NSSI professionals and consumer advocate panellists across the three Delphi survey rounds are shown in Table 2. The section headings of the Delphi questionnaire that the items were categorised into are shown in Table 3.

**Table 2:** **Participation of Delphi panellists in each round**

	**Round 1**	**Round 2**	**Round 3**
Mental health professionals	28	19	19
Consumers	33	26	25

**Table 3:** **Sections in the Delphi questionnaire**

**Section**	**Topic**
1	If the first aider finds someone injuring themselves
2	If the first aider suspects self-injury
3	Discussing self-injury
4	Alternatives to self-injury
5	Harm minimisation practices
6	Seeking help
7	What the first aider should know about self-injury
8	Adolescent specific

Pearson’s r was calculated to determine the correlations between the professional and consumer panels’ ratings. For the 191 items rated in Round 1, and the 68 items in Round 2, the item endorsement rates of the consumer panel and the professional panel were strongly correlated, with correlation coefficients of .91 and .75 respectively.

**Figure 1:**
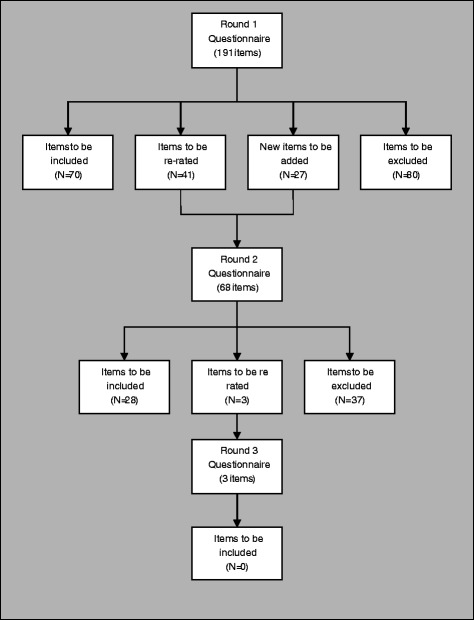
**Overview of statements throughout the 3 rounds of questionnaires.**

The inclusion, exclusion and re-rating rates for each round are shown in Figure 1. Ninety-eight statements were endorsed for inclusion in the first aid guidelines as the helping actions a member of the public should take to assist someone who self-injures. These statements have been incorporated into a plain language document to comprise the guidelines (see Additional file 1).

## Discussion

This study aimed to re-develop first aid guidelines for assisting someone who engages in non-suicidal self-injury. Searches of the available literature were conducted to find helping actions. These were then included in a survey and rated by expert panellists regarding their importance for inclusion in the guidelines. Statements that were consensually rated as important by both panels comprised the re-developed guidelines.

### Comparison with original guidelines for suicidal thoughts and behaviours

In comparison with the original 2008 version of the guidelines, some significant similarities and differences have been noted. In the current study, no items were endorsed in the ‘harm minimisation’ section of the Delphi questionnaire, while only one item was endorsed in the earlier study (to ensure first aid supplies are accessible to the person). This section involved actions the first aider could take in order to reduce the occurrence of self-injury, and to care for the injuries in order to reduce further harm (for example, through cuts getting infected). This suggests that expert opinion in the area of harm minimisation has not changed greatly over the past 6 years, and that it is an area that is difficult to provide advice in, with no consensus on whether first aiders should remove self-injury instruments, provide sterile instruments or ensure medical first aid supplies are available.

The re-developed guidelines differ from the previous version, not just through their currency, but also in providing a more comprehensive set of first aid actions than those originally developed in 2008 [25]. The Delphi survey used in the current study comprised 220 novel statements that were rated by the panellists over the three rounds. This is a substantial increase of 141 statements on the 79 statements that comprised the original Delphi questionnaire. Compared with the 30 statements endorsed in the original guidelines, the re-development saw 68 more statements endorsed, with the re-developed guidelines totalling 98 endorsed statements. Of the 30 statements endorsed in the original version of the guidelines, 18 of these were re-endorsed. The substantial increase in endorsed statements makes the recommended helping actions more specific and detailed, reducing uncertainty around how to carry out an action.

More detail is particularly notable in the sections of the guidelines on identification of alternatives to self-injury and seeking professional help, giving more specific information as to when, where and how these actions should be carried out. Roughly 10 extra statements were endorsed from each of these sections compared with the original guidelines. This includes more direction in determining when and how to engage professional help, as well as a variety of alternative coping strategies the first aider can suggest and encourage the person to use (for example, offering information about alternate coping strategies or encouraging them to talk to someone about their feelings).

Three additional sections were added to the questionnaires, these being ‘discussing self-injury’, ‘adolescent-specific’ and ‘what the first aider should know’. The section detailing how to discuss self-injury with the person who self-injures is the biggest section in the re-developed guidelines, comprising 43 recommended actions. These include advice on when and how to talk to the person, what to talk about, what to avoid saying to them (such as promising to keep their self-injury a secret), as well as how to be an active listener.

The inclusion of adolescent-specific statements provided recognition that adolescents engaging in NSSI may need more guidance and support compared to an adult. This would allow for the person providing the first aid to tailor their assistance in an age-appropriate manner. However, only one statement on seeking professional help was endorsed specifically for adolescents. This involved helping the adolescent map out a plan of action for seeking help and offering to go along with them to an appointment with a mental health professional. The other additional section outlined what information the first aider should know to place them in the best position to provide assistance. This included knowledge of the signs that indicate someone may have been self-injuring, the reasons why people self-injure, as well as clarification of the myths and the facts about NSSI.

This increase in both the number and detail of the recommendations included in the guidelines can be considered a reflection of the increase in NSSI prevention expertise, research and its subsequent literature that has developed over the past 6 years since the development of the original guidelines. This in itself highlights the importance of conducting revisions of guideline documents, as much change in the literature and expert opinion can occur across the short span of a few years.

A further difference to the original guidelines is that carers of persons who had engaged in self-injury were not included as expert panellists in the current study. Typically in MHFA guideline development, three expert panels have been recruited: mental health professionals, consumers and carers. Kelly et al. [25] attempted to represent the expertise and experiences of those who have cared for self-injuring persons, but found carers difficult to recruit. Due to the small number of carers recruited in the previous Delphi study, their endorsement ratings were combined with those of the consumer panel in the end. Because of these previous difficulties in recruitment, the working group made the decision to not have a carer panel in this Delphi study.

### Comparison between ratings of professional and consumer panels

Overall, professionals and consumers rated items similarly. Correlations between the panels’ ratings were high across Rounds 1 and 2. This indicates that both panels generally agreed on what helping actions should be included in the guidelines, and what should be excluded. This included agreement about the importance of the first aider responding in a calm and understanding manner, and acting with empathy towards the person who self-injures. Both professionals and consumers also agreed on the importance of the first aider knowing the myths and facts about NSSI.

However, while the ratings were quite similar, some notable differences were evident in the ratings assigned to statements between the professional and consumer panels. Ratings differed in the type of support each panel expected the first aider to provide. For example, while 87% of consumers highly endorsed that the first aider should ensure that adequate first aid supplies are available if they live with the person, only 58% of professionals agreed. Similarly, 83% of consumers indicated that the first aider should offer to go along with the person to see a GP or mental health professional, compared to only 58% of professionals. Furthermore, consumers placed more importance on the helping process being consumer driven. For example, 84% of consumers highly endorsed letting the person do most of the talking and 90% endorsed letting the person remain in control over seeking help as much as possible, compared to 52% and 65% of professionals respectively.

On the other hand, professionals assigned slightly higher ratings to statements that involved the first aider ensuring the safety of the person, which places the first aider in a position to seek appropriate help with the person. For example, 96% of professionals endorsed that the first aider should not promise the person that they will keep their self-injury a secret, compared to 77% of consumers.

### Strengths

The most important strength of this study is that is has resulted in re-developed first aid guidelines for NSSI, which ensured that the guidelines contain the most current and up-to-date recommendations, reflecting the most recent recommendations in the NSSI intervention literature. In doing so, the current guidelines provide greater depth and direction to guide the administration of first aid than the 2008 guidelines. Furthermore, the larger panel sizes recruited for the guideline re-development give more stable results than those obtained in the previous Delphi study. Compared with the original guidelines, 17 more panellists participated in the first round questionnaire. This increase in panel numbers reflects the broader range of NSSI prevention expertise and experiences was drawn upon in the guideline re-development.

### Weaknesses

Despite recruiting the recommended panel sizes, there were drop-outs across the rounds of the study. Only 60% consumers participated in all three questionnaire rounds, with 65% of professionals taking part in all three rounds. As the first survey was expected to take approximately 45 minutes to complete, the time commitment required for the first round questionnaire may have deterred panellists from participation in subsequent rounds. However, despite these drop-out rates, the recommendation of a minimum of 23 Delphi panellists [33] was reached for both panels for the rating of the majority of items which occurred in Round 1.

Furthermore, while these guidelines have included adolescent-specific statements to allow first aiders to tailor their assistance in a developmentally appropriate manner, the guidelines have not been developed to incorporate cultural differences. The application of the guidelines to non-western cultures and ethnic minorities is an area requiring further investigation and consultation with NSSI experts from these cultural and ethnic backgrounds.

## Conclusion

Through the Delphi process, the first aid guidelines for NSSI have been updated to ensure they are current and include the most recent and appropriate helping actions. This re-development has added depth to the previous version of the guidelines, giving more guidance in discussing self-injury with the person, seeking professional help, suggesting and encouraging alternative coping strategies, and providing assistance to adolescents who self-injure, as well as important background information and facts about NSSI. These guidelines will now be made freely available for download on the Mental Health First Aid website and inform future revisions of MHFA training courses.

## Additional file

Additional file 1:
**Items endorsed into and rejected from the guidelines, and the corresponding round consensus was reached.**
