# Correction to: Re-development of mental health first aid guidelines for non-suicidal self-injury: a Delphi study

**DOI:** 10.1186/s12888-021-03402-z

**Published:** 2021-11-18

**Authors:** Anna M. Ross, Claire M. Kelly, Anthony F. Jorm

**Affiliations:** 1 Melbourne School of Population and Global Health, The University of Melbourne, Level 4, 207 Bouverie St, Parkville, Victoria 3010 Australia; 2 Mental Health First Aid Australia, Level 6, 369 Royal Parade, Parkville, Victoria 3052 Australia; 3 School of Psychology, Deakin University, Burwood, Victoria 3125 Australia

**Correction to: BMC Psychiatry 14:236:2014**

**Doi: 10.1186/s12888-014-0236-5**

Following the publication of the original article [1], the authors identified discrepancies between Fig. 1 and Additional file 1.

**Fig. 1:**
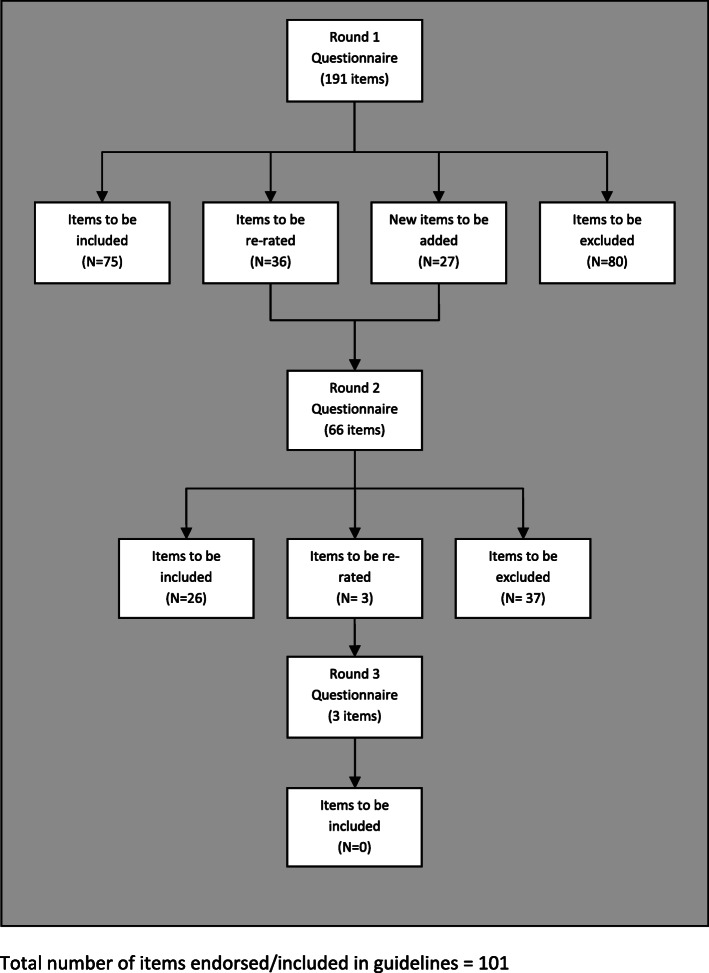
Overview of statements throughout the 3 rounds of questionnaires. Total number of items endorsed/included in guidelines = 101

The original article [1] has been corrected.

## Supplementary Information

**Additional file 1.** Items endorsed into and rejected from the guidelines, and the corresponding round consensus was reached.
